# Medical Department of Michigan University

**Published:** 1852-11

**Authors:** 


*Medical Department of Michigan University.*

WE find the following in a letter to the *Democratic Press*, of this city, from a correspondent at Ann Arbor. We hope and believe that the statements are not correct. We are aware that a few individuals would be glad to have a chair of homœopathy established in the university, but we do not believe the *people* of Michigan are making any such effort. The fact, however, that homœopaths desire any such unnatural alliance with the *science* of medicine, indicates that their faith in the system is as weak as their diluted tinctures.

At present, there are 145 students in the medical department, and the number is constantly increasing. There are at least ten of the number who are open advocates of the homœopathic practice, and twice that number who regard the system favorably. The people of Michigan are making an effort to establish a chair of homœopathy in this institution, and there is a fair prospect of their being successful. A petition to that effect is in circulation, and is being generally signed. Its chief opposition comes from the "regular practice."

Yours, MEDICUS.

